# The Impact of a Plant-Based Diet on Gestational Diabetes: A Review

**DOI:** 10.3390/antiox10040557

**Published:** 2021-04-02

**Authors:** Antonio Schiattarella, Mauro Lombardo, Maddalena Morlando, Gianluca Rizzo

**Affiliations:** 1 Department of Woman, Child and General and Specialized Surgery, University of Campania “Luigi Vanvitelli”, 80138 Naples, Italy; antonio.schiattarella@unicampania.it (A.S.); maddalena.morlando@unicampania.it (M.M.); 2 Department of Human Sciences and Promotion of the Quality of Life, San Raffaele Roma Open University, 00166 Rome, Italy; mauro.lombardo@uniroma5.it; 3 Independent Researcher, Via Venezuela 66, 98121 Messina, Italy

**Keywords:** inflammation, oxidative stress, gestational diabetes, plant foods, antioxidants, Mediterranean diet

## Abstract

Gestational diabetes mellitus (GDM) represents a challenging pregnancy complication in which women present a state of glucose intolerance. GDM has been associated with various obstetric complications, such as polyhydramnios, preterm delivery, and increased cesarean delivery rate. Moreover, the fetus could suffer from congenital malformation, macrosomia, neonatal respiratory distress syndrome, and intrauterine death. It has been speculated that inflammatory markers such as tumor necrosis factor-alpha (TNF-α), interleukin (IL) 6, and C-reactive protein (CRP) impact on endothelium dysfunction and insulin resistance and contribute to the pathogenesis of GDM. Nutritional patterns enriched with plant-derived foods, such as a low glycemic or Mediterranean diet, might favorably impact on the incidence of GDM. A high intake of vegetables, fibers, and fruits seems to decrease inflammation by enhancing antioxidant compounds. This aspect contributes to improving insulin efficacy and metabolic control and could provide maternal and neonatal health benefits. Our review aims to deepen the understanding of the impact of a plant-based diet on oxidative stress in GDM.

## 1. Introduction

Gestational diabetes mellitus (GDM) represents a challenging pregnancy complication in which women present a state of glucose intolerance that is diagnosed for the first time during pregnancy. It has been estimated that 5 to 7% of pregnancies are complicated by diabetes, and almost 80% is GDM [1,2,3]. Diagnosis of GDM is achieved by the 75 g oral glucose tolerance test, although thresholds vary among different health and diabetes associations [4].

GDM has been associated with various obstetric complications, such as polyhydramnios, preterm delivery, shoulder dystocia, and increased rates of cesarean delivery [5]. Moreover, the fetus could suffer from congenital malformations, macrosomia, neonatal respiratory distress syndrome, hypoglycemia, and intrauterine death [5]. GDM presents a challenging diagnosis, and its management can be difficult. Ethnicity is a risk factor for the development of GDM, as the incidence of GDM is increased among Hispanic and African women [6]. Obesity, a family history of type 2 diabetes (T2D), and a prior history of GDM could also increase the risk of GDM.

There is increasing evidence that GDM presents a genetic component like T2D and aggregates within families [7]. Women with a diabetic sibling had an 8.4-fold increased risk of GDM [8]. In addition, specific gene variants of melatonin receptor 1B (MTNR1B), transcription factor 7-like 2 (TCF7L2), and insulin receptor substrate 1 (IRS-1) have been found to be associated with GDM [9,10]. It has been speculated that a high sugar intake and increased weight gain during pregnancy might be responsible for an inflammatory pathway that impacts on the onset of insulin resistance [11,12]. Findings from other studies correlated heme iron levels with GDM but not non-heme iron derived from plant-based foods such as grains, vegetables, and seeds [13,14,15].

The first line therapy for GDM is based on a lifestyle approach with a low glycemic diet and an increase in physical activity. If these measures are not effective in reaching the desired glycemic control, a drug approach with insulin can be started. In view of the above, the role of the diet is crucial during pregnancy. Additionally, other pregnancy-related diseases such as hypertension and fetal growth restriction could be affected by dietary patterns [16].

Several studies have been performed about the effects of different dietary patterns on GDM, but the findings are not conclusive [17,18]. A plant-based diet could represent a suitable option for preventing inflammation through a wide range of antioxidant-rich foods [19]. The literature suggests that a high intake of vegetables, fruits, grains, fish, and legumes, according to the Mediterranean diet (MedDiet), presents a low glycemic pattern and may lower the risk of GDM in a low-risk population [20,21,22].

Zamani et al. revealed that food quality can also impact on GDM. In fact, following unhealthy dietary patterns seems to increase the risk of GDM [23].

The balance between oxidant and antioxidant molecules also represents a pivotal aspect of treating the inflammatory state in GDM [24]. Oxidative stress is characterized by a critical imbalance between antioxidant defenses and reactive oxygen species (ROS) [25]. Hyperglycemia could facilitate ROS production, thus creating an inflammatory state and increasing insulin resistance.

Vascular impairment is another mechanism involved in GDM related to oxidative stress [26]. Chronic exposure to ROS could lead to the increased production of mediators that drive stress-signaling pathways and cause potential tissue damage to key target organs, such as the vasculature and pancreas [26]. Tumor necrosis factor-α (TNF-α) has a pivotal role in insulin resistance as its concentrations are raised in GDM [27]. 

Despite the few data regarding the role of different dietary patterns in the onset and development of GDM, we aim to assess and deepen the understanding of the impact of a plant-based diet on oxidative stress in GDM.

## 2. The Impact of a Plant-Based Diet on Gestational Diabetes

The term “plant-based” presents a wide definition as it could either partially include a limited amount of foods derived from animals or include only plant foods such as fruits, vegetables, and legumes [28]. 

A MedDiet is characterized by food derived from plants without a complete exclusion of animal-source foods. In contrast, both vegetarian and vegan diets exclude meat, chicken, and fish, and a vegan diet additionally excludes dairy and eggs. 

A plant-based diet is rich in fibers, magnesium, potassium, and antioxidants but presents a lower intake of saturated fatty acids. 

**Figure 1:**
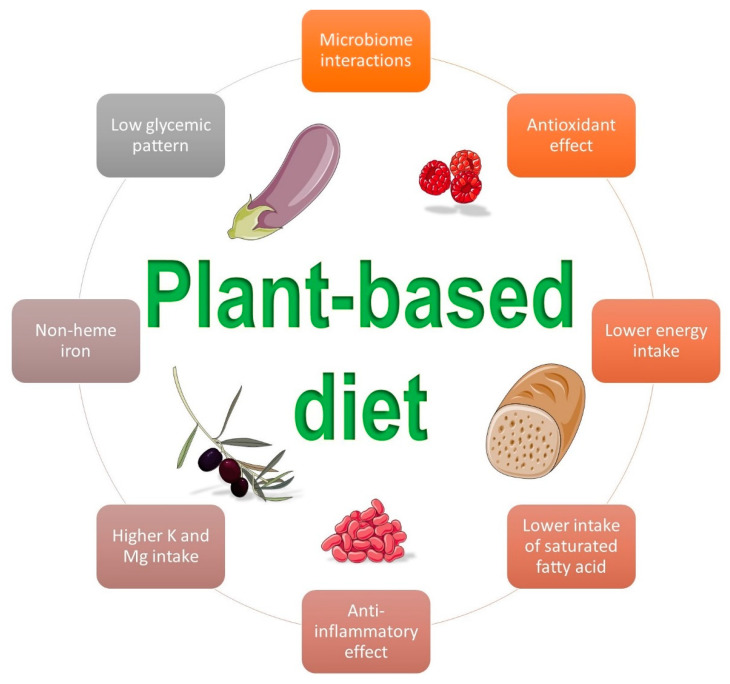
Beneficial influences of a plant-based diet on gestational diabetes mellitus (Images by smart.sevier.com).

A plant-based diet can exert its role in the prevention of GDM via multiple mechanisms of action. Figure 1 summarizes some beneficial effects of this dietary pattern.

Most dietetic associations agree that a well-planned vegetarian diet presents an adequate amount of nutrients and is helpful for the prevention and treatment of several diseases [29,30]. Although plant-based diets are associated with an increased risk of nutritional deficiencies such as vitamin B12, the available evidence also supports a well-planned vegetarian or vegan diet as a safe option during pregnancy and lactation [31,32]. However, they require strong awareness and monitoring to achieve a balanced intake of all the key nutrients. In a retrospective study of 1419 women, Kesary et al. found that a maternal vegan diet might act as a protective factor from maternal weight gain but that it also increases the occurrence of lower birth weight in the neonate [19]. In a different study, a calorie-restricted vegetarian diet was found to increase insulin sensitivity compared to a conventional diabetic diet over 24 weeks of gestation [33]. Moreover, physical activity along with a low calorie diet had a positive effect on oxidative stress marker levels. A vegetarian diet was also reported to reduce intramyocellular lipid concentrations and visceral fat, favorably impacting on insulin sensitivity and enzymatic oxidative stress markers [33].

Zulyniak et al., in 2017, analyzed 3997 full-term Canadian mothers and found that a plant-based diet was associated with increasing numbers of neonates with a low birth weight in women of Caucasian ethnicity, while, at the same time, the same dietary pattern was associated with increasing numbers of neonates with a higher birth weight in women of Asiatic ethnicity living in Canada [34].

The MedDiet and its role in the prevention of GDM has also been the subject of several investigations. A prospective study by García de la Torre et al. analyzed 1066 normoglycemic women before 12 gestational weeks following a MedDiet with extra-virgin olive oil (EVOO) and pistachio supplementation and found that GDM incidence and maternal–fetal outcomes were lower than in the control group [35]. In a different prospective study including 1076 pregnant women adhering to a MedDiet pattern, better glucose tolerance and a decreased incidence of GDM was highlighted [36].

A case-control study of 299 pregnant women affected by GDM found that a high adherence to the MedDiet before pregnancy was strongly associated with a decreased risk in GDM, suggesting a dose-dependent fashion [37]. 

Mak et al. performed a prospective cohort study of 1337 Chinese pregnant women and did not find a significant association between the risk of GDM and early pregnancy dietary patterns. However, the authors found that a high protein–low starch diet decreased the risk for GDM among obese women [38].

In a different study, the Dietary Approach to Stop Hypertension (DASH) was found to be effective to prevent GDM in 200 pregnant women [39]. The DASH, which was created to lower blood pressure, emphasizes a lower sodium intake and prefers foods rich in potassium, magnesium, and calcium [39]. 

Jali et al. analyzed 325 pregnant women undergoing screening for GDM and found out that 52 (16%) presented GDM. Particularly, authors have revealed an increased prevalence of GDM in patients following a non-vegetarian diet compared to a vegetarian diet (65.5% vs. 38.5%) [40]. 

Another Indian study analyzed 5100 pregnant women and revealed that non-vegetarianism was associated with an increased risk of developing GDM [41]. 

According to the available evidence, several possible mechanisms could explain the beneficial effects of a plant-based diet on GDM: the presence of fibers and vegetable proteins, a higher intake of antioxidants, a lower intake of saturated fat, and a higher intake of non-heme iron [42,43].

Table 1 summarizes the available studies and the main findings concerning the link between a plant-based diet and GDM.

## 3. The Role of Insulin Sensitivity in Pregnancy

Pregnancy is characterized by metabolic and immunological changes and by a physiological state of insulin resistance. All these aspects are reversible after delivery [46]. Women who develop GDM usually recover after pregnancy but up to 55% of them will develop T2D during subsequent years [47]. GDM presents similar characteristics to T2D, such as the risk factor of obesity, age, and ethnicity. These findings could suggest that GDM may help in the detection of a genetic susceptibility to develop T2D given the hormonal shift caused by pregnancy. 

Several studies have deepened the understanding of the molecular changes involved in insulin resistance in the third trimester of pregnancy, but few data are available about the hormonal interactions during the first and second trimesters [48,49,50].

It seems that during the first trimester of pregnancy insulin secretion increases to promote adipose tissue storage. In the third trimester, there is instead a shift through increased insulin resistance, a rise in free fatty acid (FFA) serum concentrations, and, therefore, a reduction of adipose tissue storage [51,52]. Given the above, insulin exerts a reduced capacity to control lipolysis in the late part of pregnancy, which is more significant in women affected by GDM [51]. 

Insulin resistance is characterized by a reduction in the ability of the liver, adipose tissue, and muscle to intake adequate glucose. Insulin normally binds the insulin receptor provoking the phosphorylation of the β-subunit receptor and insulin receptor substrate-1 (IRS-1) on at least six tyrosine residues [46]. 

IRS-1 phosphorylation provokes the binding and phosphorylation of the regulatory subunit p85α of phosphatidylinositol 3-kinase (PI 3-kinase) to IRS-1 [53]. P85α acts as a positive enhancer of the insulin pathway and seems to be relevant for activating glucose transporters (GLUT) in lipid cells [54]. Particularly, levels of the p85α subunit are increased in skeletal muscle and lipid cells in pregnant women with GDM compared to obese non-pregnant women [51].

Pregnant women showed a reduction of IRS-1 expression, and this finding could explain the reduced activity of insulin on its pathway [47]. 

Friedman et al. found that insulin resistance in pregnancy correlates with a reduced activity of IRS-1 tyrosine phosphorylation [47]. This aspect is related to a reduced expression of IRS-1. Particularly, women affected by GDM present a reduced activity of tyrosine phosphorylation of the β-subunit receptor and an impairment in the glucose transporter [47]. 

Another mechanism involved in insulin resistance is the increase in IRS-1 serine phosphorylation. This aspect seems to impair the action of insulin on its receptor, the IRS-1 tyrosine phosphorylation, and, therefore, glucose cellular intake [55]. 

These alterations could represent an initial insulin resistance and also an increased risk to develop T2D during life [47]. 

Recent studies analyzed the role of apolipoprotein A-1 (ApoA1), the main lipoprotein associated with high density lipoprotein (HDL), in insulin resistance. However, despite the finding of a relationship between HDL and ApoA1 and the activation of pancreatic β-cell function, there were no conclusive findings on this aspect [56,57]. 

Adipokines, such as adiponectin and leptin, are cytokines secreted by fat cells that are involved in the regulation of insulin secretion as well as in fetal growth [58,59]. 

Adiponectin improves glucose intake in skeletal muscle cells and decreases glucose secretion in the liver [60]. Moreover, the serum levels of adiponectin decline during pregnancy in both obese and lean women. Given the above, adiponectin could be considered as an insulin-sensitizing agent [60].

Leptin, produced mainly by lipid cells, presents a serum concentration proportional to the adipose tissue percentage [58]. Leptin is also produced by the human placenta, and its receptors were found in both the maternal and fetal surfaces, suggesting an autocrine or paracrine effect on placenta functioning [61].

Thyroid function anomalies could influence glucose homeostasis and insulin resistance in women affected by diabetes [62]. New evidence has suggested that higher concentrations of thyroid-stimulating hormone (TSH) and free triiodothyronine (FT3) as well as an elevated FT3:thyroxine (FT4) ratio could suggest an increased risk of GDM [63].

Other authors have found an association between reduced amylase levels and GDM [64]. This relationship, still defined in patients affected by T2D, could be considered as a predicting factor for GDM [64,65].

So far, the mechanisms by which female hormones such as estrogens and progestins act on insulin resistance during pregnancy is not completely understood.

As it is increased during normal pregnancy, progesterone might play a crucial role in this process. Progesterone inhibits the phosphatidylinositol-3-kinase-mediated pathway by blocking the expression of IRS-1 [43]. Moreover, it suppresses insulin-induced GLUT-4 translocation [43]. 

Estrogens, particularly 17beta-estradiol, seem to present a complementary role along with progesterone regarding insulin sensitivity. Estrogens are involved in storing and exporting triglycerides from liver, thus impacting on insulin sensitivity [66]. Several studies have analyzed the impact of hormone replacement therapy on improving liver function and the risk of non-alcoholic fatty liver disease in women affected by T2D [67,68].

Human placental lactogen (hPL), secreted by syncytiotrophoblast cells, promotes insulin resistance [69]. It is associated with glucose cell intake, glycogen storage, and oxidation [69]. At the same time, it sustains the storage of energetic supplies for the fetus, thus increasing maternal hyperglycemia.

### The Effects of a Plant-Based Diet on Insulin Sensitivity

The impact of diet on glucose homeostasis and therefore on insulin sensitivity has been investigated by several studies in the literature [70,71].

Plant-based diets have spread worldwide in the last few years, according to evidence of such diets preventing T2D, cardiovascular diseases, and cancer [72].

Vegetables and fruits present an important amount of fiber, which is known to reduce gastric emptying and, therefore, lower the glycemic response [73]. Fiber could also positively impact on inflammatory markers and fat storing in the liver [74].

Unsaturated fatty acids, mainly found in olive oil and nuts, improve insulin sensitivity through a reduction of serum lipids and ameliorate the inflammatory response of adipose tissue [75]. 

Kahleova et al. revealed that a plant-based diet enhances postprandial incretin and insulin secretion [76].

In another study, Bligh et al. found that plant-rich meals significantly increase the serum levels of glucagon-like peptide-1 (GLP-1), a hormone that augments the secretion of insulin, and peptide YY (PYY), while the gastric inhibitory peptide (GIP) level is lowered [77].

## 4. The Link between Microbiota and Diet

The intestinal microbiota includes trillions of microbic cells and represents a critical aspect of human wellbeing as it seems to modulate the risk of several chronic diseases, such as inflammatory bowel and immune diseases, T2D, cardiovascular disease, and cancer. 

The gut microbiota is involved in different activities: protection against pathogenic bacteria and systemic immunomodulation and production of metabolites from foods [78,79]. 

Moreover, it is fundamental for T-cell and B-cell differentiation in the gut [78]. 

The microbiota starts to develop immediately after the vaginal passage during birth and many factors contribute to its development, such as age, diet, and genes. Moreover, it has been speculated that the vertical transmission from the mother to the offspring represents another key aspect of this process [80]. 

The human gut microbiota is characterized by five bacterial groups: Firmicutes, Bacteroides, Proteobacteria, Actinobacteria, and Verrumicrobia. However, the predominant bacteria are Firmicutes and Bacteroides. The microbiota could be considered as an endocrine organ, as it produces metabolites and molecules that impact on several functions [79]. Particularly, through fermentation, gut bacteria produce short-chain free fatty acids that exert immunomodulatory activity [81]. 

During pregnancy, hormones modulate the composition of gut microbiota: from the first to the third trimester, there is an increase in Proteobacteria and Actinobacteria and an increase in heterogeneity, also called beta diversity [82]. A decreased richness of bacterial populations, or alpha diversity, represents the opposite condition in the last part of the pregnancy [82]. 

In the third trimester, there is an increase in gut inflammation and hyperglycemia is promoted [82]. This condition has been considered as an adaptative function in order to augment energy storage for fetus development [82]. 

Another important aspect is the reduction of short-chain-FFA-producing bacteria [82]. This change represents a critical point that permits a rise in the levels of T regulatory cells, a subpopulation of the immune system that preserves tolerance to self-antigens and forestalls autoimmune diseases [82]. In particular, T regulatory cells might prevent maternal rejection of the fetal allograft [83]. 

The colonization by the uterine microbiota of the placenta and the amniotic fluid is a novel and relevant concept. This condition could explain how a mother’s microbiota colonizes the fetus [84,85]. Moreover, the immune tolerance of pregnancy might disclose the lack of an inflammatory activity against microbial cells in the placenta and the amniotic fluid [84]. 

The placenta seems to present a reduced microbiota colonization, particularly of non-pathogenic bacteria such as Proteobacteria [86]. This microbiota has been found to be similar to the oral cavity microbiota and could reveal the long-term link between periodontal disease and preterm birth [86]. An impairment of this colonization could also explain the development of intrauterine infections [86]. 

Other authors have underlined the correlation between bacterial DNA in amniotic fluid and the rise in immune cells [87]. However, other researchers have revealed opposite findings [88].

Given the above, pregnancy is characterized by a change in the gut microbiota that could probably contribute to the development of GDM [89]. Several studies have deepened the connection between the microbiota and dietary patterns, therefore speculating a target therapy.

*Prevotella copri* and *Bacteroides vulgatus* represent bacteria that might alter insulin homeostasis, whereas *Bacteroides* and *Staphylococcus aureus* are more expressed in obese women than in lean women [85,90]. Other researchers found that in women affected by GDM there was a reduction of Firmicutes species, while the alpha and beta diversity were similar to the control group [91]. Moreover, a different composition in the oral, vaginal, and gut microbiota was found in women affected by GDM compared to healthy pregnancies [92]. Similar findings were revealed by Bassols et al. about the placenta microbiota: in women with GDM, there were less bacteria from *Pseudomonadales* and *Acinetobacter* and increased inflammatory expression [93]. In particular, Acinetobacter seems to induce the expression of IL-10—a cytokine with an anti-inflammatory effect and that is involved in the stimulation of T cells and mast cells [93]. 

Several studies have analyzed the correlation between the microbiota and insulin resistance, but the available evidence is still scarce. Despite this consideration, diet is able to modulate the microbiota composition in a few days and it could represent an intriguing option to prevent the development of GDM.

### The Effects of a Plant-Based Diet on Microbiota

A potential beneficial effect of a plant-based diet is the dietary modulation of the gut microbiota. Protein and insoluble fibers impact on the gut microbiota by modulating the immune system and the inflammatory pathways [94]. A diet high in fibers decreases Firmicutes species and increases Bacteroidetes ones, improving short-chain fatty acid production [94]. These changes seem to reduce the incidence of inflammatory diseases and modulate the immune system [94]. Accumulating evidence underlines the impact of the diet on the microbiota. In particular, Barrett et al. revealed that women in early pregnancy and following a vegetarian diet present a different gut microbiota as compared to an omnivorous diet, with a higher abundance of bacteria producing short-chain fatty acids (SCFAs) but without any impact on GDM [44]. At the same time, a diet low in fiber and with a high glycemic intake was proven to increase the risk for GDM in a prospective study of 13,110 patients [45]. 

## 5. Oxidative Stress and Insulin Resistance

Oxidative stress remains an important factor in the development of insulin resistance. It represents a normal aspect of a healthy pregnancy, although an abnormal consumption of antioxidants could impair several energetic pathways. Moreover, this aspect is fairly recognized as a critical factor for several pregnancy diseases [95].

Oxidative stress is characterized by a disbalance between antioxidant defenses and oxidant production. The latter category involves ROS radicals, such as superoxide radical (O^2−^), nitric oxide radical (NO), and hydroxyl radical (˙HO−), and non-radicals, such as hydrogen peroxide (H_2_O_2_) and peroxynitrite (ONOO^−^) [25].

Conversely, antioxidant processes involve several enzymes, such as superoxide dismutase (SOD), catalase, thioredoxin, and glutathione peroxidase (GTX) [25]. Non-enzymatic compounds comprise glutathione, ascorbate, and α-tocopherol.

The main causes of oxidative disbalance are the mitochondrial overproduction of ROS, due to high glucose levels and lipid excess, and nicotinamide adenine dinucleotide phosphate (NADPH) oxidase’s increased action through angiotensin II (ANG II) receptors [96]. 

Several studies have claimed higher levels of oxidative stress and inflammatory markers such as xanthine oxidase, lipid peroxides, malondialdehyde (MDA), 8-isoprostane, TNF-α, and IL-10 in women affected by GDM [97,98] [97,98].

Moreover, systemic insulin resistance, along with oxidative stress, has been correlated not only to an impairment of liver, muscle, and fat tissue but also to central nervous system deterioration [99]. Some authors have revealed that higher levels of insulin modify the blood–brain barrier through a reduced expression of insulin receptors [100]. This mechanism reduces the permeability of insulin through the central nervous system [100].

Particularly, chronic oxidative stress could impact on neuroplasticity and neuronal survival [99]. Insulin plays a critical role in neuronal functioning by participating in neuronal metabolism, as GLUT-4 has been found on neuronal membranes [101]. However, insulin exerts its metabolic effects through the autonomic nervous system on the liver to suppress glucose production or increase triglyceride secretion [101,102]. 

An Indian research group found that TNF-α is significantly associated with pre-eclampsia in GDM women while increased serum levels of uric acid, IL-8, and TNF-α were associated with maternal–fetal adverse outcomes [103]. In contrast, antioxidants such as serum bilirubin, GTX, and SOD positively correlated with pregnancy outcomes [103].

Jamilian et al. performed a randomized clinical trial (RCT) to analyze the effects of a supplementation of omega-3 fatty acids and vitamin E in 60 women affected by GDM [104]. The outcomes revealed an increase in total antioxidant capacity, MDA, and NO levels and decreased fetal hyperbilirubinemia incidence [104].

Other studies have discovered that in mammalian skeletal muscle cells oxidant stress activates a serine kinase p38 mitogen-activated protein kinase (p38 MAPK) pathway linked to a decrease in insulin sensitivity [96]. 

Other findings regarding the role of the renin–angiotensin system (RAS) and oxidative stress were presented by Wei et al. They observed that ANG II interferes with the phosphorylation of IRS-1 tyrosine and Protein kinase B (Akt) serine thus impairing insulin-dependent GLUT-4 translocation. This mechanism has been found to be prevented by blocking NADPH oxidase [105]. ANG II can also act through the nuclear factor-κB (NF-κB) pathway to interfere with insulin resistance [106]. Moreover, Akaishi et al. found that the ANG II receptor antagonist reduced ROS concentrations induced by high glucose levels in human renal mesangial cells [107].

Several antioxidants have been studied in insulin resistance. Alpha-lipoic acid (ALA) can reduce the formation in vitro of advanced glycation end products (AGEs), which are markers of oxidative damage and associated with impaired insulin action [108].

Pyridoxamine (PYR), a B6 vitamin family compound, is involved in different metabolic pathways that directly inhibit AGE formation thanks to the ability of trapping reactive carboxyl intermediates, although few and inconclusive data are available on humans [109,110].

Oxidative stress has also been implicated in the development of diabetic complications such as retinopathy, nephropathy, peripheral neuropathy, and heart disease [111]. It alters gene expression and tissue functionality. Increased ROS production by the mitochondria in endothelial cells has a pivotal role in microvasculature damage [96].

### The Effects of a Plant-Based Diet on Oxidative Stress

Environment and lifestyle concur to achieve a healthy pregnancy. Moreover, intrauterine development seems to be crucial for preventing several diseases, such as cardiovascular and renal diseases, T2D, and neurological impairment [21]. This particular condition has been studied and defined as “early life programming” [112].

Given the above, the role of the maternal diet, along with sleep quality, physical activity, and avoiding smoking and pollutants, is crucial for the fetus. New evidence has been raised about the benefit of dietary patterns, such as a plant-based diet, on maternal outcomes in pregnancy [113]. Women following a plant-based diet seem to incur in a reduction in pregnancy hypertensive disorders and GDM thanks to the increased intake of fibers [45,114].

Plant-based diets represent a new approach for promoting health. However, the term “plant-based” has a fairly broad definition, as it could either partially include a limited amount of foods derived from animals or include only plant foods such as fruits, nuts, grains, vegetables, and legumes [28].

Several studies have deepened the relationship between this kind of diet and the reduction of the incidence of chronic diseases [115,116,117]. 

A plant-based diet may increase antioxidant status and nitric oxide bioavailability and decrease ROS, homocysteine serum levels, blood pressure, hyperglycemia, lipids, and even atherosclerosis [118]. Oxidative stress is triggered by an imbalanced redox state caused by mitochondria dysfunction, the overproduction of ROS, or a defective antioxidant system. Several authors have correlated chronic oxidative stress and inflammation with obesity. A healthy dietary pattern is a powerful tool to achieve redox homeostasis. Food and drink with adequate antioxidant compounds, physical activity, and stress management are fundamental aspects for the achievement of a healthy weight and to promote weight loss in obese and overweight people [119]. In view of the above, plant-based diets have spread worldwide in the last few years, according to the knowledge that they might improve the overall quality of life and lead to a healthier status by not only preventing coronary heart disease, cancer, and type 2 diabetes but also ameliorating menopausal complaints in women [73,120,121]. 

The most widespread plant-based diet is the MedDiet, a dietary pattern associated with a reduced risk of heart disease, metabolic syndrome, diabetes, and cancer [120]. 

A MedDiet is characterized by food derived from plants such as fruits, vegetables, oils, grains, legumes, beans, and nuts with no absolute exclusion of animal-source foods. In contrast, both vegetarian and vegan diets exclude meat, poultry, and fish, and a vegan diet additionally excludes animal products such as dairy and eggs. A plant-based diet is rich in fibers, dietary nitrates, and cardioprotective micronutrients, such as magnesium and potassium, and antioxidants and low in saturated/trans fats. In contrast, animal foods are typically much lower in nitrates, magnesium, potassium, and antioxidants. 

There is observational and interventional evidence that a plant-based diet high in antioxidants, micronutrients, nitrates, and fibers and low in saturated and trans fats may decrease the incidence and severity of several diseases [118,121]. 

## 6. Conclusions

Our review highlights that a healthy plant-based diet might favorably impact on the onset of GDM. GDM remains a pregnancy complication influenced both by genetic and inflammatory factors. An imbalanced redox state could act as a trigger to alter insulin sensitivity, and a healthy dietary pattern such as a plant-based one represents a suitable option to improve the intake of antioxidant compounds. However, further studies, especially RCTs, are required to deepen the current knowledge on the interactions between diet and the redox state.

## Figures and Tables

**Table 1:** Main findings of the studies concerning the impact of a plant-based diet on gestational diabetes mellitus.

Reference	Type of Study	Main Outcome	Number of Participants	Event	Definition of Plant-Based Diet
Arora et al., India [41]	Observational, cross-sectional	An increased risk of developing GDM was associated with a non-vegetarian diet	5100 women	The prevalence of GDM was 35% using WHO 2013 criteria	Vegetarian diet
Barrett et al., Australia [44]	RCT	A vegetarian diet in early pregnancy increased the presence of short-chain fatty acid bacteria producers without any influence on GDM risk	9 following a vegetarian diet and 18 an omnivorous one	Microbiome alpha diversity was similar, while beta diversity was reduced, in vegetarians	Vegetarian diet
De Filippis et al., Italy [42]	Observational	An increased consumption of plant foodstuffs based on a MedDiet was associated with beneficial microbiota improvements	51 vegetarians, 51 vegans, and 51 omnivores	Positive correlation between consumption of vegetables and short-chain fatty acids, *Prevotella*, and Firmicutes in the gut microbiome	MedDiet
García de la Torre et al., Spain [35]	Observational, prospective	Following a MedDiet with EVOO and pistachio supplementation before 12 gestational weeks showed a lower GDM incidence and better maternal–fetal outcomes	932 women	The incidence of GDM was lower in the intervention group than in the controls (RR 0.81)	MedDiet
Izadi et al., Iran [39]	Observational, case-control	Adherence to the DASH and MedDiet was associated with a reduced risk for GDM	200 women with GDM and 260 without GDM	A higher adherence to DASH was related to 71% reduced risk for GDM	DASH and MedDiet
Jali et al., India [40]	Observational, cross-sectional	Non-vegetarian pregnant women showed an increased risk for glucose intolerance	325 women: 202 vegetarian and 123 non-vegetarian	52 women (16%) presented GDM. An increased prevalence of GDM in patients following a non-vegetarian diet compared to a vegetarian diet (65.5% vs. 38.5%)	Vegetarian diet
Kahleova et al., Czech Republic [33]	RCT	A low calorie vegetarian diet improved insulin sensitivity	37 following a vegetarian diet and 37 following a conventional diabetic diet	A vegetarian diet improved adipokine levels and oxidative stress markers compared to a conventional diabetic diet over 24 weeks	Vegetarian diet
Karamanos et al., Mediterranean countries [36]	Observational, prospective	Adhering to a MedDiet pattern decreased the incidence of GDM	1076 women	The incidence of GDM was lower in subjects with better adherence to the MedDiet (8.0% vs. 12.3%)	MedDiet
Kesary et al., Israel [19]	Observational, retrospective	A vegan diet is a protective factor from maternal weight gain but increased the risk for a lower birth weight	234 vegans, 133 vegetarian, and 1052 omnivores	A vegan diet in pregnancy was associated with a lower birth weight centile compared to omnivores (42.6 ± 25.9 vs. 52.5 ± 27.0; *p* < 0.001)	Vegan and vegetarian diet
Mak et al., China [38]	Observational, prospective	Following an early pregnancy dietary pattern did not significantly increase the risk of GDM in patients. However, a high protein–low starch diet was associated with a decrease in risk for GDM among obese women	1337 women	199 women (14.9%) developed GDM	Plant-based and a high protein–low starch pattern diet
Olmedo-Requena et al., Spain [37]	Observational, case-control	A high adherence to a MedDiet before pregnancy was strongly associated with a decreased risk in GDM	291 with GDM and 1175 without GDM	A high MedDiet adherence was associated with lower GDM risk (aOR 0.61; *p* = 0.028), while a very high MedDiet adherence was more strongly associated (aOR 0.33; *p* = 0.005)	MedDiet
Zhang et al., USA [45]	Observational, prospective	A low fiber and high sugar intake diet increased the risk for GDM	13,100 women	758 with GDM. Each 10-g/day increment in total fiber intake was associated with a 26% reduction in GDM risk	Diet rich in fiber
Zulyniak et al., Canada [34]	Observational, prospective	A plant-based diet was associated with lowering the birth weight for women of Caucasian ethnicity and increasing it in Asiatic women living in Canada	3997 women	The plant-based diet was inversely associated with birth weight (β = −67.6 g per 1-unit increase; *p* < 0.001)	Plant-based diet

## Abbreviations

AGE	Advanced Glycation End Products
Akt	Protein kinase B
ALA	Alpha-lipoic acid
ANG II	Angiotensin II
ApoA1	Apolipoprotein A-1
CRP	C-Reactive Protein
DASH	Dietary Approaches To Stop Hypertension
EVOO	Extra-Virgin Olive Oil
FFA	Free fatty acid
FT3	Free Triiodothyronine
FT4	Thyroxine
GDM	Gestational Diabetes Mellitus
GLUT	Glucose Transporter
GTX	Glutathione Peroxidase
HDL	High density lipoprotein
HPL	Human Placental Lactogen
I-κB	Kappa b inhibitor
IL	Interleukin
IRS-1	Insulin Receptor Substrate 1
MDA	Malondialdehyde
MedDiet	Mediterranean Diet
MTNR1B	Melatonin Receptor 1B
NAPDH	Nicotinamide Adenine Dinucleotide Phosphate
NF-κ	Nuclear Factor Kappa
NO	Nitric Oxide
P38 MAPK	Serine Kinase P38 Mitogen-activated Protein Kinase
PI 3-kinase	Phosphatidylinositol 3-kinase
PYR	Pyridoxamine
RAS	Renin–angiotensin system
RCT	Randomized Controlled Trial
ROS	Reactive Oxygen Species
SCFA	Short-chain Fatty Acid
SOD	Superoxide Dismutase
T2D	Type 2 Diabetes
TCF7L2	Transcription Factor 7-like 2
TNF-α	Tumor Necrosis Factor-α
TSH	Thyroid-stimulating hormone
